# Antifungal Activity of Native *Trichoderma afroharzianum* and *Arcopilus cupreus* Against *Neopestalotiopsis rosae* Causing Strawberry Root and Crown Rot in Mexico

**DOI:** 10.3390/jof12060388

**Published:** 2026-05-28

**Authors:** Karla Jenifer Juárez-Cruz, Alfredo Jiménez-Pérez, Federico Castrejón-Ayala, Gabriela Trejo-Tapia, Lauro Soto-Rojas, Alma Rosa Solano-Báez, Guillermo Márquez-Licona

**Affiliations:** 1 Centro de Desarrollo de Productos Bióticos, Instituto Politécnico Nacional, Yautepec 62731, Morelos, Mexico; kjuarezc2302@alumno.ipn.mx (K.J.J.-C.); aljimenez@ipn.mx (A.J.-P.); fcastrej@ipn.mx (F.C.-A.); gttapia@ipn.mx (G.T.-T.); 2 Posgrado en Fitosanidad-Entomología y Acarología, Colegio de Postgraduados, Campus Montecillo, Montecillo, Texcoco 56264, Estado de Mexico, Mexico; rojo@colpos.mx

**Keywords:** *Neopestalotiopsis rosae*, *Trichoderma afroharzianum*, *Arcopilus cupreus*, strawberry root and crown rot, morphology, phylogeny, pathogenicity, strawberry diseases

## Abstract

Root and crown rot of strawberry is an emerging disease of concern in Mexico. Like other soil-borne diseases, it has spread widely due to the vegetative propagation of this crop. This study aimed to evaluate the in vitro antagonistic activity of four native *Trichoderma* strains and one strain of *Arcopilus cupreus* against a population of *Neopestalotiopsis rosae*, as a potential biological control alternative. Thirty-five commercial strawberry fields across the three main strawberry-producing states were sampled, yielding 103 fungal isolates. Nearly 70% of the recovered isolates belonged to the *Neopestalotiopsis* genus. Sixteen representative isolates were identified as *N. rosae* based on morphology and multilocus sequence phylogeny (ITS, *β-tub*, *tef1*-α) and confirmed as pathogenic through pathogenicity tests. Four native *T. afroharzianum* isolates recovered from the rhizosphere of healthy strawberry plants were identified by multilocus sequence analysis (*rpb2*, *tef1-α*). All *T. afroharzianum* isolates exhibited antagonistic activity in dual-culture assays, inhibiting mycelial growth by 71–73%, surpassing the effectiveness of the commercial fungicide cyprodinil + fludioxonil (average inhibition 50%). In contrast, the *A. cupreus* isolate recovered from a native medicinal plant showed an average inhibition of 38%. These results highlight native *T. afroharzianum* as a promising sustainable alternative for integrated management of strawberry root and crown rot in Mexico.

## 1. Introduction

The strawberry (*Fragaria* × *ananassa;* Rosaceae) is among the most widely cultivated and consumed berry species [1]. Mexico is the fifth-largest producer of strawberries worldwide, with an average annual production of 641,552 tons [2]. Michoacán (411,927 tons), Baja California (137,424 tons), and Guanajuato (107,920 tons) are the three main producing states [3]. This crop faces numerous phytosanitary challenges, with fungal diseases being the predominant factor deterring productivity. This problem affects production from the seedling stage onward and reduces fruit quality. In Mexico, strawberry seedlings are produced through vegetative propagation, which complicates the acquisition of certified, pathogen-free plants, primarily because nurseries lack effective means of producing healthy plants and implementing phytosanitary practices to reduce pathogen inoculum. To reduce production costs, standard methods include reusing trays, ineffective disinfection practices, and the use of non-sterile substrates (personal communication with producers and affected growers).

During the strawberry production stage, primary soil-borne diseases include anthracnose caused by *Colletotrichum acutatum*, charcoal rot caused by *Macrophomina* sp., crown and root rot caused by *Phytophthora cactorum*, *P. citricola*, and *Pythium*, leaf and crown rot caused by *Neopestalotiopsis* spp., Fusarium wilt caused by *Fusarium oxysporum*, Verticillium wilt caused by *Verticillium dahliae*, and root rot caused by *Rhizoctonia* spp. [4,5]. However, the most significant in recent years is root and crown rot caused by *Neopestalotiopsis rosae*, first reported in Mexico in 2020 [6]. This pathogen, originally reported as a phytopathogen on *Rosa* sp. plants in New Zealand in 1998 [7], was first detected in Egypt [8], affecting strawberry crops. From there, it has spread to other countries where strawberries are cultivated, including China [9], Mexico [6], Turkey [10], USA [11], Paraguay [12], India [13], Germany [14], and Italy [15]. In Mexico, the disease has been exacerbated by the lack of certified, disease-free plant material, which has led to its spread across most strawberry-growing regions, and by unknown levels of soil inoculum. Additionally, the lack of cultural practices that reduce inoculum sources, such as harvesting crop residues, contributes to the problem.

Infection by *N. rosae* primarily causes root and crown rot in strawberries; however, it can also infect other parts of the plant, with specific symptoms in each tissue type. On leaves, the disease starts with small brown spots that spread across the leaf blade, leading to leaf blight, during which black pycnidia and conidia form. Infected petioles develop sunken, dark-brown lesions, causing the leaves to wilt. Likewise, the pathogen can infect the fruit, creating slightly sunken, light-brown spots where black pycnidia develop. The interior of infected crowns displays areas of intense red surrounded by dark-brown margins, which ultimately leads to plant death. On the roots, rot symptoms begin with the development of large, dark-brown areas [6,16].

The main strategy for managing the disease is the use of synthetic fungicides; thus, multiple studies have evaluated the efficacy of various fungicidal compounds [5,6,8,17,18]. However, there is growing concern about environmental issues, public health risks, and the development of resistance to fungicides used in agriculture [19]. Biological control offers an alternative management strategy to synthetic fungicides [20]. Among the organisms used in this approach, *Trichoderma* is one of the most extensively studied fungal genera due to its diverse mechanisms of action against other fungi [21]. For example, *T. asperellum* has demonstrated a significant in vitro inhibitory effect against isolates of *N. clavispora*, the causal agent of strawberry root rot in China [20]. Additionally, *T. reesei* and *T. harzianum* have demonstrated significant inhibition of *N. chrysea* mycelial growth, which causes leaf spots on strawberries in Bangladesh [22]. In field experiments, *T. asperellum* and *T. koningiopsis* have proven effective against *N. rosae* [5]. In contrast, the use of *Arcopilus cupreus* as a biological control agent against plant pathogenic fungi has not been extensively studied. However, this species shows potential as a biocontrol agent due to its production of antifungal metabolites, mycoparasitic activity, competition for space and nutrients, or a combination of these mechanisms [23]. *A. cupreus* inhibits the growth of *Phytophthora* spp. [24] and has demonstrated antifungal activity against *Botrytis cinerea*, *Pythium ultimum*, and *Rhizoctonia solani* [25].

Although evaluations of biological products and synthetic fungicides for disease management have been conducted in Mexico [5], this disease continues to be a serious limiting factor in strawberry production, as no integrated management program has yet been developed that is both accessible and adaptable to the diverse management practices and microclimatic conditions present in production areas. In addition, the continuous evolution and adaptation of phytopathogenic fungi in response to human-driven crop management practices [26,27] underscore the ongoing need to discover new, more effective, and sustainable management strategies. Therefore, the present study aimed to evaluate the in vitro antagonistic activity of four native *Trichoderma* strains and one of *A. cupreus* against a population of *N. rosae***,** the causal agent of root and crown rot in strawberries in Mexico, as a potential biological control alternative. We hypothesized that native antagonistic fungi isolated from Mexican strawberry agroecosystems could effectively suppress different *N. rosae* genotypes.

## 2. Materials and Methods

### 2.1. Recollection of Diseased Strawberry Plants

**Figure 1:**
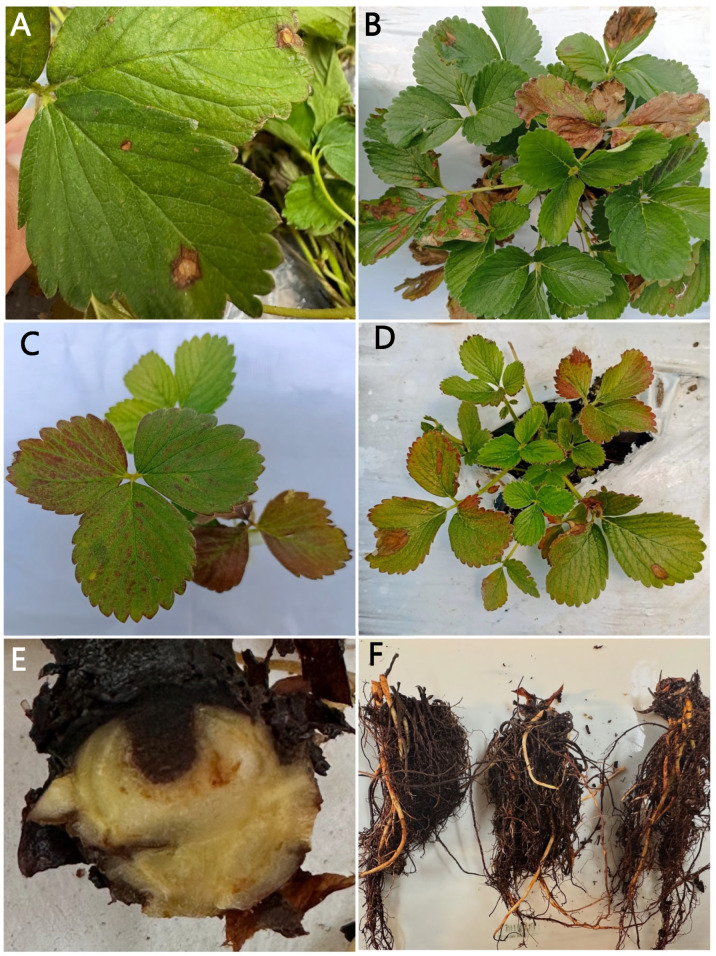
Symptoms caused by *Neopestalotiopsis rosae* on strawberry plants. (**A**) Leaf spots. (**B**) Leaf blight. (**C**) Reddish-purple coloration on leaves. (**D**) Necrosis on the edges of the leaves. (**E**) Crown rot. (**F**) Root rot.

In October 2023, diseased strawberry plant samples were collected in Michoacán (Central-Western Region), Guanajuato (Central-Western Region), and Baja California (Northwest Region), guided by observations of characteristic foliar symptoms induced by *Neopestalotiopsis* (Figure 1A,B). Only plants showing symptoms of root and crown rot were included. Sampling was conducted across diverse production systems and levels of technification to capture the broadest possible range of pathogen diversity associated with strawberry root and crown rot. Plants were initially selected based on reddish-purple leaf discoloration (Figure 1C,D), a symptom commonly associated with the early stages of root and crown rot. Upon excavation, plants exhibited extensive root necrosis with dark-brown discoloration (Figure 1E), and their crowns showed intense reddish internal coloration, surrounded by necrotic margins (Figure 1F). A total of 35 samples were collected, each comprising 10 plants exhibiting initial disease symptoms. The collected samples were placed in airtight plastic bags and transported in a cooler (10 ± 2 °C) to the Phytopathology Laboratory at the Centro de Desarrollo de Productos Bióticos, Instituto Politécnico Nacional (CEPROBI-IPN), located in Yautepec, Morelos. During the next two production cycles (2024–2025), strawberry plants with symptoms of root and crown rot were collected, and only the frequency of isolation at the genus level was determined.

### 2.2. Isolation, Purification, and Preservation of the Fungi Associated with the Disease

The samples were washed under running water until most of the soil and organic matter were removed. To isolate the fungi associated with the disease, 5-mm tissue fragments from the transition zone between healthy and diseased tissue were collected from each sample with a scalpel. The recovered tissue was surface-disinfected with 1.2% sodium hypochlorite for 2 min, then rinsed three times with sterile distilled water. After disinfection, the tissue was placed on sterile paper towels and allowed to dry for 2 h. Four tissue fragments were placed in each Petri dish containing potato dextrose agar (PDA; BD Bioxon^®^, Cuautitlán Izcalli, Estado de Mexico, Mexico), and the plates were incubated at 25 °C for 48 h. Colonies were then transferred to fresh PDA and incubated at 25 ± 2 °C for 10 days [28]. To obtain axenic cultures, isolates were purified by the single-spore technique. Conidia from the fungal colonies were suspended in 1 mL of sterile distilled water in a microtube and vigorously agitated. From this conidial suspension, 200 μL were plated onto water agar (AA; BD Bioxon^®^, Cuautitlán Izcalli, Estado de Mexico, Mexico), and the plates were incubated at 25 ± 2 °C for 24 h. From each isolate, one germinating conidium was selected and transferred to PDA. Plates were incubated at 25 °C under a 12-h light/dark photoperiod for 7 days [28]. Of the isolates recovered from strawberry plants, 16 representative isolates were selected based on collection sites and morphotypes. Each isolate was preserved in triplicate in 10% glycerol and stored at 4 °C. The isolates were deposited under IPN 10.0227—IPN 10.0242 in the Culture Collection of Phytopathogenic Fungi of the CEPROBI-IPN.

### 2.3. Cultural Characterization

Descriptions of colony morphology and reproductive structures were based on colonies grown on PDA and incubated at 22 ± 2 °C under a 12-h light/dark photoperiod for 7 days [7]. The growth kinetics of each isolate were determined on PDA at 22 ± 2 °C by marking the colony margin every 24 h until the fungus completely covered the plate. Daily growth was measured with a vernier Traceable (Fisherbrand, Pittsburgh, PA, USA), and the mycelial growth rate was calculated using the formula of Zervakis et al. (2001) [29]. Semi-permanent slides of the reproductive structures were prepared with lactic acid as the mounting medium and sealed with a wax ring. Observations were made using an Axio Imager A2 compound microscope (Zeiss^®^, Oberkochen, Ostalb, Germany). Images were captured with an Axiocam ICc5 camera (Zeiss^®^, Oberkochen, Ostalb, Germany), and measurements were made using the ZEN software lite version (Zeiss^®^, Oberkochen, Ostalb, Germany). For morphometric characterization, only three representative isolates (IPN 10.0227, IPN 10.0239, and IPN 10.0242) were selected. The length and width of conidia (*n* = 100) and basal cells (*n* = 50) were measured, along with the length of apical and basal appendages and the number of appendages.

### 2.4. Molecular Identification

Genomic DNA was extracted from the sixteen selected isolates using colonies grown on PDA at 22 ± 2 °C under a 12 h light/dark photoperiod for 7 days, following the protocol for the “DNeasy Plant Mini Kit” (Qiagen, Hilden, Nordrhein-Westfalen, Germany). The quality and quantity of the extracted DNA were assessed using a spectrophotometer, NanoDrop 2000 (Thermo Scientific, Wilmington, DE, USA). The recovered DNA was stored at −20 °C. The ITS region and partial sequences of the β-tubulin (*β-tub*) and translation elongation factor 1-alpha (*tef1-α*) genes were amplified using the primer pairs ITS5 + ITS4 [30], T1 + Bt2b [31,32], and EF1-728f + EF-2 [33,34], respectively. The PCR reactions were conducted in a total volume of 50 μL, including 25 μL of PCR Master Mix 2X (Promega Corporation, Madison, WI, USA), 2.5 μL of each primer (10 μM), 16 μL of nuclease-free water, and 4 μL of the DNA template. The PCR cycling parameters were as follows: ITS region: initial denaturation at 95 °C for 3 min; followed by 40 cycles of 95 °C for 30 s, 55 °C for 50 s, and 72 °C for 60 s; with a final extension at 72 °C for 7 min. *β-tub*: initial denaturation at 95 °C for 3 min; followed by 40 cycles of 94 °C for 30 s, 55 °C for 50 s, and 72 °C for 60 s; with a final extension at 72 °C for 7 min. *Tef1-α*: initial denaturation at 95 °C for 5 min; followed by 40 cycles of 94 °C for 30 s, 52 °C for 30 s, and 72 °C for 30 s; with a final extension at 72 °C for 7 min. PCR products were visualized by electrophoresis on a 1% agarose gel and purified using the MiniElute Gel Extraction Kit (Qiagen^®^, Hilden, Nordrhein-Westfalen, Germany) according to the manufacturer’s instructions. The PCR products were sequenced in both directions by Macrogen^®^ (Seoul, Republic of Korea). Sequence quality was verified by inspecting the electropherograms using the 4Peaks software v1.8 (https://nucleobytes.com/4peaks/index.html, accessed on 2 March 2025). Consensus sequences were assembled using UGene v52.1 [35] and compared against the GenBank database using the BLASTn tool (https://blast.ncbi.nlm.nih.gov/Blast.cgi, accessed on 5 March 2025) to determine isolate identity at the genus level. The consensus sequences were deposited in the GenBank database of the National Center for Biotechnology Information (NCBI) (Table 1). A reference phylogeny [7] was used to construct the phylogenetic matrix.

Sequences from species included in the phylogeny were downloaded from the NCBI nucleotide database and aligned with our sequences using the MAFFT v7 online service [36]. The alignments were trimmed in MEGA 12 [37] and then concatenated in Mesquite 4.01 [38] for the multigene analysis. For phylogenetic reconstruction, a Bayesian Inference (BI) analysis was performed using MrBayes 3.2.7 [39], applying the nucleotide substitution models GTR + I (ITS), GTR + G (*β-tub*), and HKY + I (*tef1-α*), all identified by the Akaike Information Criterion (AIC) in jModelTest 2.1.10 [40]. The Markov Chain Monte Carlo (MCMC) process was run for 1 × 10^7^ generations to estimate posterior probabilities, with trees sampled every 1000 generations and 25% discarded during the burn-in phase. Additionally, a Maximum Likelihood (ML) phylogenetic analysis was performed using RAxML 8.2.12 via the raxmlGUI 2.0 interface [41], under the GTR + I + G substitution model. Node support was assessed using 1000 rapid bootstrap replicates. Phylogenies were visualized and edited using FigTree (http://tree.bio.ed.ac.uk/software/figtree/, accessed on 14 March 2025).

### 2.5. Pathogenicity Tests

Healthy strawberry plantlets (var. Sayualita), 60 days old, were transplanted from trays into 1 L plastic pots containing sterile substrate (peat moss mixed with perlite at a 5:1 *v*/*v* ratio). The plants were acclimatized for 15 days in a greenhouse at 25 ± 5 °C. Agronomic management involved daily irrigation and weekly fertilization with a mixed solution containing Raizal 400 (Arysta LifeSciences, Saltillo, Coahuila, Mexico) and Gro-Green (Gro-Green Campbell, Gomez Palacio, Durango, Mexico), each at 1 g × L^−1^. Each plant was irrigated with 250 mL of this solution.

The inoculum for the sixteen isolates was increased by culturing on PDA plates and incubating in the dark at 25 ± 2 °C for 15 days. Inoculum recovery was performed by scraping the pionotal masses with a sterile spatula and transferring them into a 1% Tween 60 solution. The inoculum was homogenized using a handheld blender, applying 10-s pulses for a total of one minute. The inoculum concentration was adjusted to 1 × 10^6^ conidia/mL using a Neubauer chamber (Marienfeld, Meno-Tauber, Baden-Wurtemberg, Germany). Inoculation was performed by injecting 5 mL of the conidial suspension into the crown of six strawberry plants for each isolate [42]; the plants were then covered with a transparent plastic bag and incubated at 25 ± 2 °C for 7 days. As a control, six plants were treated the same way, with the inoculum replaced by sterile distilled water. The experimental design followed was a completely randomized design. For the evaluation of disease severity in pathogenicity tests, a scale was developed based on crown inoculation symptoms previously reported by other authors [6,43,44], consisting of the following levels: 0 = 0% healthy plant, 1 = 20% yellowing of the oldest leaves, 2 = 40% stunted growth, 3 = 60% wilting of young leaves and leaf necrosis, 4 = 80% death of the oldest leaves while central leaves remain green, 5 = 100% plant death. The disease severity percentage was calculated using the following formula [45].

$$P=\frac{\sum_{i=0}^{5}n_{i}\times V_{i}}{5\times N}\times 100$$

where:

*P* = severity percentage.

$n_{i}$ = number of plants in category *i*.

$V_{i}$ = numerical value of category *i*.

*N* = sample size.

Re-isolations were made from the inoculated plants. The recovered fungi from artificially inoculated symptomatic plants were identified based on morphological characteristics.

Data from the pathogenicity tests were analyzed using a one-way analysis of variance (ANOVA), with the *N. rosae* isolate as the main source of variation. When significant differences were detected, treatment means were compared using Bonferroni-corrected pairwise comparisons at a significance level of *p* < 0.05. Prior to analysis, data were examined to verify compliance with the assumptions of normality and homogeneity of variance. All statistical analyses were conducted in R version 4.5.3, with a significance threshold of 5%. The experiment was repeated twice with similar results.

### 2.6. Collection, Isolation, and Identification of Native Biocontrol Agents

Native *Trichoderma* isolates were obtained from four localities in the state of Michoacán with no history of strawberry cultivation. At each locality, a 1-kg rhizosphere soil sample from healthy strawberry plants was collected. For *Trichoderma* isolation, soil samples were dried to constant weight, homogenized, and 1 g of soil was transferred into a test tube containing 9 mL of sterile distilled water. From this suspension, three additional serial dilutions were prepared. From the final dilution, 200 µL were spread onto Trichoderma Specific Medium (TSM) [46,47]. Plates were incubated at 25 ± 2 °C for 96 h, after which colonies growing on the medium were recovered. Purification was performed using the single-spore culture technique, and isolates were preserved in triplicate in 10% glycerol. In total, 25 *Trichoderma* isolates were obtained; only the four with the highest antifungal activity in preliminary tests were selected, identified, and evaluated in this study. The selected isolates were deposited under the accession numbers MBP1–MBP-4 in the Collection of Native Microorganisms with Biotechnological Potential (MBP) of the CEPROBI-IPN.

Descriptions of colonies and morphology of the *Trichoderma* isolates were based on isolates grown on PDA and SNA incubated at 25 ± 2 °C with a 12 h light/dark photoperiod for 3 weeks [48]. The growth kinetics of each isolate were determined on PDA by measuring mycelial growth every 24 h [29]. Measurements of the reproductive structures were made using the same microscopes used for the strawberry fungi.

For molecular identification of the *Trichoderma* isolates, DNA extraction from pure cultures was performed using the same kit employed for the isolates recovered from the strawberry. The ITS region and partial sequences of the RNA polymerase II second-largest subunit (*rpb2*) and translation elongation factor 1-alpha (*tef1-α*) genes were amplified using the primer pairs ITS5 + ITS4 [30], RPB2-5F2 [49]/fRPB2-7CR [50], and EF1/EF2 [34]. Amplicon visualization and purification, as well as sequencing and sequence processing, were performed as previously described. The multigene phylogenetic analysis used to determine the phylogenetic species of *Trichoderma* was conducted with a reference phylogeny (Table 2) [48], following the previously described methodology for alignment, trimming, and concatenation. For phylogenetic reconstruction, Bayesian Inference (BI) was conducted using the nucleotide substitution models GTR+I (*rpb2*) and HKY+I (*tef1-α*). The ITS region was used to corroborate the isolate’s genus-level identity, but it was not included in the phylogenetic analysis. The Markov Chain Monte Carlo (MCMC) procedure was run for 1 × 10^6^ generations to estimate posterior probabilities, sampling trees every 1000 generations and discarding the first 25% as burn-in. In addition, Maximum Likelihood (ML) analysis was performed under the GTR+I substitution model, with node support evaluated using 1000 rapid bootstrap replicates. The resulting phylogenies were visualized and edited with FigTree.

The *Arcopilus cupreus* isolate (IPN 10.0154) was previously obtained from *Baccharis conferta* Kunth [51], a native medicinal plant commonly found in the Iztaccihuatl–Popocatepetl National Park. Although this fungus was reported to cause mild leaf spot symptoms in its host, it was serendipitously found to inhibit the growth of phytopathogenic fungi through antibiosis, prompting its inclusion in the present study to assess its potential as a biocontrol agent against *N. rosae*.

### 2.7. Antagonism Tests

In dual-culture assays, four *Trichoderma* sp. isolates (MBP1–MBP4) and one *Arcopilus cupreus isolate* (IPN 10.0154) were confronted with sixteen *Neopestalotiopsis* isolates. Each *Neopestalotiopsis* isolate was inoculated along one edge of the Petri dish, while the biological control agent was placed along the opposite edge, with approximately 6 cm between the colonies. Three replicates were established per treatment, and the inoculated plates were incubated at 25 ± 2 °C until the non-confronted fungus (control) completely covered the plate. In addition, a commercial fungicide, cyprodinil + fludioxonil (375 + 250 g kg^−1^), formulated as Switch^®^ 62.5WG (Syngenta S.A. de C.V., Ciudad de Mexico, Mexico), was evaluated using the poisoned food technique on PDA at a concentration of 1 μg mL^−1^ [52]. The experiment was established under a completely randomized factorial design, with antagonist treatment and *Neopestalotiopsis* isolate as factors, using three replicates per treatment. Colony growth was recorded every 24 h, and daily radial growth was measured with a digital caliper. Mean values of the percentage of radial growth inhibition (*PRGI*) were calculated using the formula described by Dennis and Webster (1971) [53]:

$$PRGI=\frac{R1-R2}{R1}\times 100$$

where:

*R*1 = diameter of the control colony.

*R*2 = diameter of the colony in confrontation with the antagonistic organism.

Prior to analysis, data were examined to verify compliance with the assumptions of normality and homogeneity of variance. The inhibition percentages were subjected to a two-way analysis of variance (ANOVA), and means were compared using the Holm–Sidak test in R version 4.5.3 (*p* < 0.05). The antagonistic capacity of the *Trichoderma* isolates was classified according to the criteria described by Bell et al. (1982) [54], based on observations from dual culture assays against *N. rosae* isolates.

## 3. Results

### 3.1. Isolation of Strawberry Pathogens

Sampling was conducted in 35 commercial strawberry fields across the three principal strawberry-producing states of Mexico (Michoacán, Baja California, and Guanajuato). From the processed samples, 103 fungal isolates were recovered, of which 73 (68%) belonged to the genus *Neopestalotiopsis,* 30 (28%) to *Macrophomina*, and four (4%) to *Fusarium*. This data showed that *Neopestalotiopsis* was the most frequently recovered genus from symptomatic plants across the surveyed states. However, in 2024 and 2025, a consistent increase in the recovery rate of *Macrophomina* isolates was observed, with *Macrophomina* exceeding *Neopestalotiopsis* in some localities of Michoacán and Guanajuato during the 2024 season. In 2023 samples, mixed infections were detected in 25% of processed samples, with fungi from two genera recovered from the same plant, indicating the frequent coexistence of multiple pathogens in affected fields. Of the 73 *Neopestalotiopsis* isolates obtained, 16 representative isolates were randomly selected for further studies, including 12 from Michoacán, two from Baja California, and two from Guanajuato. These isolates were subjected to morphological identification, multilocus phylogenetic analysis, pathogenicity tests, and subsequent in vitro antifungal assays using native biological control agents.

### 3.2. Cultural Characterization

**Figure 2:**
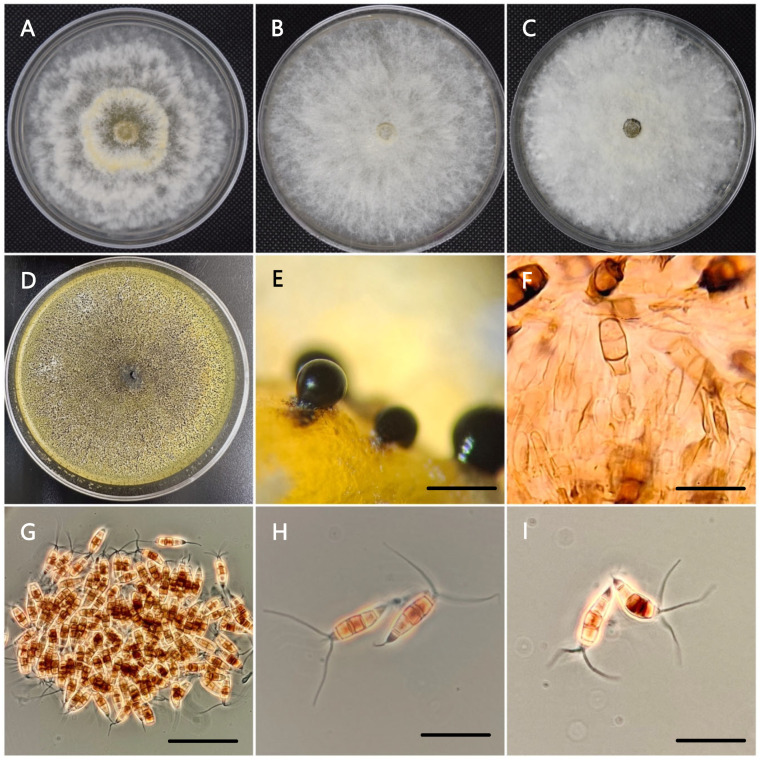
Cultural and morphological characteristics of *Neopestalotiopsis rosea* isolates recovered from crown rot symptoms. (**A**). Morphotype 1: isolate IPN 10.0227 (Michoacán). (**B**). Morphotype 2: isolate IPN 10.0242 (Guanajuato). (**C**). Morphotype 3: IPN 10.0239 (Baja California). (**D**). Pionotal masses in PDA 21 days after plate inoculation. (**E**). Close-upside view of pionotal masses in culture medium. (**F**). Conidiogenic cells. (**G**). Pionotal mass under a stereoscopic microscope. (**H**,**I**). Conidia with appendages. Scale Bars: (**E**) = 100 µm; (**F**) = 20 µm; (**G**) = 50 µm. (**H**,**I**) = 30 µm.

Colonies of all isolates grown on PDA produced abundant white, cottony mycelium with globose, solitary, semi-immersed, dark-brown to shiny pionnotal masses of the acervular type (Figure 2A–C). The reverse of the colonies exhibited a pale-yellow pigmentation. Colony margins and growth patterns varied among isolates, allowing classification into three morphotypes based on macroscopic characteristics. Pionnotal masses developed 14 days after inoculation (Figure 2D,E). Conidiogenous cells were hyaline, cylindrical, and smooth-walled, measuring 6–18 × 3–6 µm (Figure 2F). Conidia were fusiform and 5-celled, with three dark-brown median cells and separated by four dark septa. The basal cell was conical and hyaline, bearing a single appendage. The apical cell was cylindrical, hyaline, thin-walled, and smooth, with two to four tubular, filiform apical appendages. These appendages were not inserted at a single apical crest but rather individually attached at different points along the upper half of the apical cell and were unbranched. (Figure 2G–I).

Although isolates were overall morphologically similar, statistical analysis of conidial length and width separated them into two groups based on geographic origin. Isolates from Michoacán and Guanajuato produced larger conidia (22.0–37.0 × 7.5–9.5 µm) compared with isolates from Baja California (21.0–23.0 × 6.0–6.5 µm). Similarly, pionnotal masses from Michoacán (105 µm) and Guanajuato (103 µm) were substantially larger than those from Baja California (22 µm). Mycelial growth rates also differed significantly (*p* ≤ 0.05), with isolates from Michoacán and Guanajuato growing faster (6.3 mm day^−1^) than those from Baja California (4.1 mm day^−1^). Based on the observed morphological characteristics, all isolates were consistent with descriptions of the genus *Neopestalotiopsis* [7,55].

### 3.3. Molecular Identification

**Figure 3:**
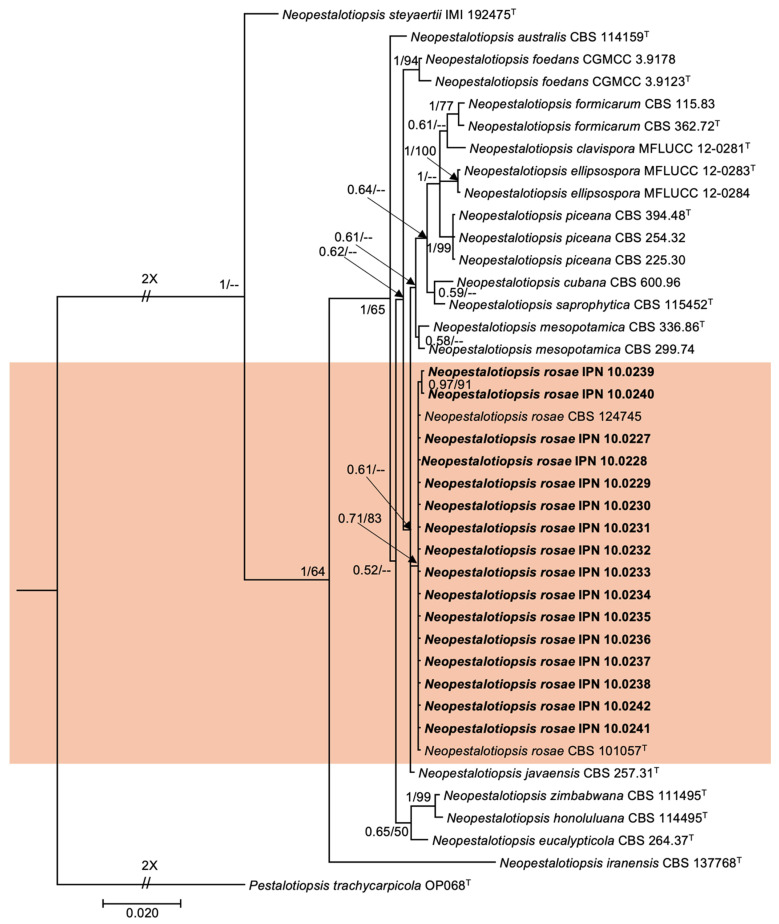
Concatenated phylogenetic tree based on Bayesian Inference and Maximum Likelihood using partial *tef1-α*, *β-tub*, and ITS sequence data from species of the genus *Neopestalotiopsis*. Posterior probabilities (>0.5) and bootstrap support values (>50%) are shown at the nodes. Type isolates are indicated with a T. Isolates from this study are highlighted in bold. The tree was rooted with *Pestalotiopsis trachycarpicola* OP068.

BLASTn searches against the GenBank database revealed that all obtained sequences showed 99–100% similarity to species within the genus *Neopestalotiopsis*. Multilocus phylogenetic analysis based on combined ITS, *tef1-α,* and *β-tub* sequences placed all 16 isolates within a well-supported clade corresponding to *N. rosae*. The isolates clustered with the ex-type strain CBS101057, with support of 0.71/83 (Posterior Probability/Bootstrap) (Figure 3). These results confirmed that the isolates recovered from strawberry plants exhibiting root and crown rot symptoms were *N. rosae*.

### 3.4. Pathogenicity Tests

All 16 *N. rosae* isolates were pathogenic. Inoculated strawberry plants developed characteristic symptoms starting 7 days after inoculation. Initial symptoms included foliar chlorosis, followed by progressive wilting and eventual plant death. Internally, affected crowns exhibited reddish-brown discoloration consistent with root and crown rot. Control plants remained asymptomatic throughout the experiment. The experiment was conducted twice, with similar results. Statistical analysis showed significant differences in disease severity among the *N. rosae* isolates (*p* < 0.05). Based on multiple comparisons of means, nine severity percentage groups were identified (Table 3). Isolates collected from Michoacán caused the highest severity, followed by those from Guanajuato, whereas isolates from Baja California resulted in significantly lower severity. Thus, the most aggressive isolate was IPN 10.0228 from Michoacán (90.05% severity), whereas the least aggressive was IPN 10.0240 from Baja California (26.70%). These results indicate substantial variability in aggressiveness among geographically distinct *N. rosae* isolates.

### 3.5. Identification of Native Biocontrol Agents

**Figure 4:**
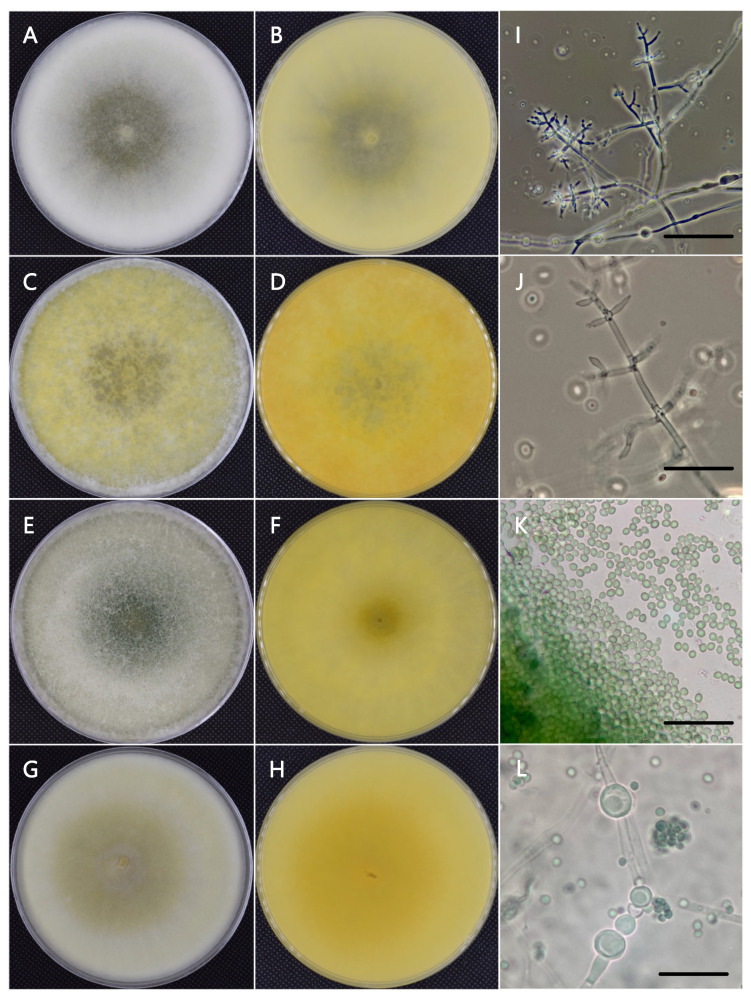
Morphology of isolates of *Trichoderma afroharzianum* recovered from the rhizosphere of strawberry plants. (**A**–**H**) Colonies on PDA after 8 days of incubation in complete darkness at 25 °C. (**A**,**B**) Isolate MBP-3. (**C**,**D**) Isolate MBP-2. (**E**,**F**) Isolate MBP-4. (**G**,**H**) Isolate MBP-1. (**A**,**C**,**E**,**G**) Front view of the colonies. (**B**,**D**,**F**,**H**) Underside of the colonies. (**I**,**J**) Conidiophores. (**K**) Conidia. (**L**) Chlamydospores. Scale Bars: (**I**) =100 µm. (**J**–**L**) = 30 µm.

After 72 h of incubation at 25 °C on PDA, *Trichoderma* colonies reached an average diameter of 58 mm, whereas on SNA medium, they reached 55 mm. At 35 °C, colony diameters averaged 40 mm on PDA and 47 mm on SNA. After 96 h of incubation at 25 °C on PDA, colonies developed abundant cottony mycelium and produced yellow pigment that occasionally diffused into the medium, whereas isolates grown on SNA produced sparse aerial mycelium. Conidiophores were pyramidal with opposite branching; the main axis and each branch terminated in a cruciform verticil bearing up to five phialides. Phialides were lageniform to ampulliform, measuring 7.0 × 3.0 µm, with a basal width of 2.0 µm and a supporting cell width of 2.7 µm. The length/width ratio of phialides was 2.6; the ratio between phialide length and supporting cell width was 3.0; and the ratio between phialide width and supporting cell width was 1.2. Conidia were ovoid to globose, smooth-walled, green, and measured 3.0 × 2.5 µm. Chlamydospore formation was frequent after 7 days of incubation in three of the four isolates (Figure 4).

**Figure 5:**
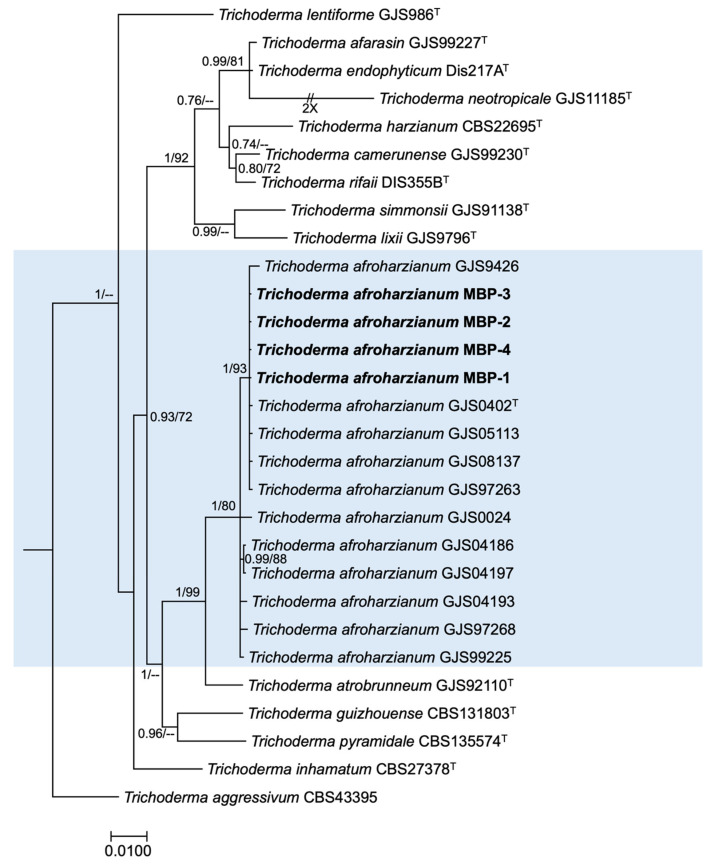
Concatenated phylogenetic tree based on Bayesian Inference and Maximum Likelihood using partial *tef1-α* and *rpb2* sequence data from species of the genus *Trichoderma*. Posterior probabilities (>0.7) and bootstrap support values (>70%) are shown at the nodes. Type isolates are indicated with a T. Isolates from this study are highlighted in bold. The tree was rooted with *Trichoderma aggressivum* CBS43395.

BLASTn searches against the GenBank database showed 99–100% sequence similarity with species of the genus *Trichoderma*. Multilocus phylogenetic analysis based on combined *rpb2* and *tef1-α* sequences placed all four isolates within the clade corresponding to *Trichoderma afroharzianum*, with strong Bayesian posterior probability and Maximum Likelihood bootstrap support (1.00/93) (Figure 5). Therefore, these isolates were confidently identified as *Trichoderma afroharzianum*.

### 3.6. Biocontrol Tests

All *Trichoderma afroharzianum* isolates (MBP-1-MBP-4) significantly inhibited the mycelial growth of the 16 *Neopestalotiopsis rosae* isolates, with mean percentages of radial growth inhibition (PRGI) values ranging from 71.0% to 73.0% compared to the untreated control. Statistical analysis showed significant differences among *T. afroharzianum* isolates. Multiple mean comparisons divided them into two groups: isolates MBP-1 (73%) and MBP-2 (72%) formed the first group, exhibiting the highest antagonistic activity, while isolates MBP-3 and MBP-4 (71%) made up the second group. In contrast, the commercial fungicide fludioxonil + cyprodinil (Switch^®^ 62.5WG) showed a mean PRGI of 50% against the same pathogen isolates, forming a third group.

When evaluated individually against each *N. rosae* isolate, 13 of the 16 *T. afroharzianum* isolates showed no significant differences in PRGI values against most pathogen isolates. However, significant differences were observed for isolates IPN 10.0232, IPN 10.0237, and IPN 10.0240. Isolate MBP-1 exhibited higher inhibition against IPN 10.0232 (78.3%) and IPN 10.0237 (77.5%) compared with isolate MBP-3 (71.2% and 70.6%, respectively). Against isolate IPN 10.0240, MBP-1 achieved 50.9% inhibition, significantly greater than isolates MBP-2, MBP-3, and MBP-4 (40.2–44.2%) (Table 4). Variation in pathogen susceptibility to *T. afroharzianum* was also evident. For example, the isolate MBP-1 inhibited isolates IPN 10.0242 and IPN 10.0241 by 81.8–82.2%, whereas inhibition of isolates IPN 10.0240 and IPN 10.0238 ranged from 50.9–51.1%. Similar patterns were observed for isolates MBP-1, MBP-3, and MBP-4, which consistently showed higher inhibition against isolates IPN 10.0241 and IPN 10.0233 but reduced activity against IPN 10.0240 and IPN 10.0238. These results indicate considerable variability in sensitivity among *N. rosae* isolates.

According to the antagonism classification by Bell et al. (1982) [54], isolates MBP-1 and MBP-2 were classified as Class I, characterized by complete overgrowth of *Trichoderma*, covering the pathogen colony. Isolates MBP-3 and MBP-4 were classified as Class II, showing overgrowth of at least two-thirds of the pathogen colony surface.

In contrast, *Arcopilus cupreus* exhibited significantly lower antagonistic activity, with a mean PRGI of 38.1% across all *N. rosae* isolates. The commercial fungicide showed a higher overall mean inhibition (70.8%) than *A. cupreus*, but displayed marked variability among pathogen isolates, with PRGI values ranging from 11.8% to 92.8%. Significant differences in fungicide sensitivity were detected for isolates IPN 10.0227, IPN 10.0228, IPN 10.0229, IPN 10.0230, IPN 10.0232, IPN 10.0233, IPN 10.0234, IPN 10.0235, and IPN 10.0238, whereas the remaining isolates did not differ significantly (Table 5). Overall, both biological and chemical treatments showed differential efficacy across *N. rosae* isolates, highlighting pronounced intra-population variability in response to control measures.

## 4. Discussion

Due to the rapid increase in global strawberry demand over recent years, the strawberry production industry has expanded, and new cultivation areas have developed [3,56]. As a leading producer, Mexico has experienced increased production as cultivation has expanded into new agricultural regions [3]. However, the movement of plant material increases the risk of pathogen dissemination. Current agricultural practices, including vegetative propagation, introduce pathogens into new production areas through infected plant material from nurseries [57,58,59,60,61]. The lack of commercial cultivars with full resistance to crown and root rot caused by *N. rosae* further exacerbates the epidemiological risk [62,63,64]. The symptoms observed in strawberry plants were similar across collection regions and consistent with previous reports describing strawberry root and crown rot symptomatology [6,16].

The cultural and morphological characteristics observed in isolates from strawberry plants showing root and crown rot symptoms, along with multigene phylogenetic analysis, enabled us to identify the causal agent as *N. rosae*. Although some nodes in the *Neopestalotiopsis* phylogeny show moderately supported values, the most comprehensive study of the genus *Neopestalotiopsis* [7] indicates that species within it are distinguishable through multigene phylogenetic reconstruction using the internal transcribed spacer (ITS), partial sequences of the genes *β-tub*, and *tef1-α*. However, these markers do not fully resolve the phylogeny of all 30 clades; thus, some clades remain poorly supported, and one clade is still classified as *Neopestalotiopsis* spp. Unfortunately, to date, higher-resolution markers are lacking to distinguish between species. The supports reported in this study align with those documented in the first report of the pathogen in Mexico [6]. Otherwise, the *Trichoderma* phylogeny, based on Chaverri et al. (2015) [48], was resolved using the amplified genes, yielding high support values.

*Neopestalotiopsis rosae* has been reported as the causal agent of strawberry crown rot in Egypt [8], Mexico [6], Taiwan [44], and China [9]. Since its first report in Mexico, this disease has rapidly spread across all strawberry-producing regions of the country, mainly due to the distribution of infected propagative material, including other pathogens, such as *Macrophomina*. This situation is largely attributable to prevailing artisanal propagation practices in Mexico, including the reuse of trays or pots previously contaminated with infested soil without proper disinfection (personal communication from technicians and affected growers).

As previously reported in Mexico [6], only the species *N. rosae* was found in the samples analyzed in the present study. The proportions of fungi isolated from strawberry plants with root and crown rot symptoms represent a first approximation to the diversity of species involved in strawberry plant mortality in the field. In the present study, nearly one-third of the plants with these symptoms belonged to the genus *Macrophomina*, indicating that this fungus is an emerging pathogen in strawberry plantations in Mexico. This highlights the epidemiological importance of *Macrophomina* as an emerging strawberry pathogen. *Macrophomina phaseolina* has been reported as the causal agent of crown rot in strawberry in Argentina [65], Chile [66], and the United States [67]. However, in Mexico, the phylogenetic species of *Macrophomina* affecting strawberry plants has not yet been confirmed through molecular methods. This fungus could become increasingly important, and research should focus on the timely development of management strategies to minimize losses caused by this pathogen.

Artificially inoculated strawberry plants showed characteristic disease symptoms seven days after inoculation. Affected plants exhibited growth retardation, yellowing of outer leaves, dark-brown lesions, and crown rot with reddish-brown discoloration, leading to plant death, symptoms previously reported for this disease [6,18]. Therefore, selection of disease-tolerant germplasm can be conducted 15 days after inoculation, when symptoms are visible. The inoculum concentration and inoculation method used in this study were appropriate, as they induced characteristic disease symptoms in a shorter time than reported by Dardani et al. (2025) [15], who documented initial symptoms appearing 10–12 days after inoculation, and even faster than reported by Sigillo et al. (2020) [42], who observed symptoms up to two months after inoculation.

Cultural and morphological characterization of the native biocontrol agents placed the four isolates within the genus *Trichoderma*, while phylogenetic species identification was conducted using multigene phylogenetic analysis of concatenated ITS sequences and partial *rpb2* and *tef1-α* sequences. This analysis confidently confirmed that the four isolates belong to the species *Trichoderma afroharzianum*, based on reference phylogeny [48].

All isolates of *T. afroharzianum* exhibited strong antagonistic activity against the 16 *N. rosae* isolates, with mean inhibition ranging from 71 to 73%. This range exceeded that reported by Olivares-Rodríguez et al. (2024) [68], who evaluated 15 *Trichoderma* spp. isolates against *Neopestalotiopsis* isolates from strawberry; only the T2 strain reached 70% inhibition, while the others showed lower levels. In this study, the interaction between strain MBP-1 of *T. afroharzianum* and isolate IPN 10.0242 resulted in a PIMG of 82.2%. The average PIMG of 71–73% is consistent with previous reports [17,68], which describe a 66.6–76% range as a significant in vitro inhibition range for *N. rosae* and *N. clavispora* recovered from strawberry by *Trichoderma* spp. The variation in the in vitro biocontrol activity of *T. afroharzianum* isolates against *N. rosae* is expected, as pathogen suppression levels can depend on the strain used and its adaptability to specific biotic and abiotic conditions [53]. *T. afroharzianum* has proven effective as a biocontrol agent against phytopathogenic fungi in wheat (*Fusarium culmorum*) [69], tomato (*Alternaria alternata*, *Botrytis cinerea*, *Fusarium oxysporum*) [70,71,72], onion (*Stromatinia cepivora*) [73], garlic (*Sclerotium cepivorum*) [74], and mustard (*Sclerotinia sclerotiorum*) [75]. Moreover, strain T22, reclassified as *T. afroharzianum*, has been used in commercial biocontrol products [48,76]. However, to the best of our knowledge, this is the first study to explore the biocontrol potential of native Mexicans *T. afroharzianum* isolates against *N. rosae*.

Microscopic observation of interactions between *T. afroharzianum* and *N. rosae* isolates revealed that mycoparasitism was the primary mechanism underlying the biocontrol effect. Because the isolates were able to grow rapidly, coil around and parasitize the pathogen’s hyphae, lyse them, and occupy space and resources, thereby limiting the growth of *N. rosae*, we can conclude that the *T. afroharzianum* isolates exhibit microparasitism and competence. Furthermore, the yellow coloration observed in the culture media in which *T. afroharzianum* grew indicates that these isolates produce compounds that inhibit the growth of *N. rosae* isolates. Therefore, the isolates likely produce compounds with antibiotic, toxic, and lytic capacities. These mechanisms not evaluated in the present study cannot be ruled out, including the production of hydrolytic enzymes, antibiosis (antifungal and volatile compounds), competition for space and nutrients, and the induction of host plant defense mechanisms previously reported for *T. afroharzianum* [70,72,73,75]. These findings demonstrate the potential of native *T. afroharzianum* isolates as biological control agents. In dual-culture assays, inhibition exceeding 70% is commonly used as a benchmark for selecting promising *Trichoderma* isolates for development as biocontrol agents [77,78,79].

*Arcopilus cupreus* is characterized by the abundant production of yellow, orange, or red exudates (secreted products) [80], a feature observed in both dual cultures and controls in the present study. The production of metabolites was evidenced by the presence of inhibition halos in encounters with *N. rosae*. Although *A. cupreus* showed moderate antagonistic activity, its inhibition levels were lower than those of *T. afroharzianum*. Acting through mechanisms such as production of antifungal metabolites, mycoparasitism, and competition for space and nutrients, enabling control of a broad range of phytopathogenic fungi [23]. Previous reports indicate antifungal activity of *A. cupreus* against *Phytophthora* spp. recovered from mandarin roots, achieving PIMG values of 62.5–73.2% [24]. Additionally, other species of this genus have demonstrated antifungal activity, such as isolate YZXR of *A. aureus*, which inhibited up to 80% of the growth of *Fusarium fujikuroi*, the causal agent of leaf spot in *Polygonatum odoratum*. Nevertheless, further research is needed to fully assess the potential of this genus as a biological control agent.

The commercial fungicide Switch^®^ 62.5WG (Fludioxonil + Cyprodinil) showed an average PIMG of 50%, with isolate IPN 10.0230 being the most sensitive (92.8% PIMG) and isolate IPN 10.0240 the least sensitive (11.8%). In contrast, Rebollar-Alviter et al. (2020) [6] reported 100% PIMG when evaluating the same fungicide against two *N. rosae* isolates obtained from strawberry crowns. These results highlight pathogen variability and the importance of conducting population studies that capture this diversity and relate isolate responses with geographic origin, to avoid generalized management recommendations, given the broad variability observed among isolates in the present study. Overall, the marked variability observed in both biological and chemical control responses emphasizes the need for population-based management strategies in Mexican strawberry production systems. Integrated disease management programs incorporating locally adapted biological agents may provide a more reliable alternative to confront the adaptive capacity of phytopathogenic fungi to intensive agricultural practices and stop relying on synthetic fungicides. Laboratory evaluation of biocontrol agents using dual-culture assays is a standard method for assessing the mechanisms by which these agents act against pathogens. These tests allow rapid comparison of many isolates and selection of the best ones. However, they capture only some mechanisms in the controlled interaction, not the full range of interactions that occur in the rhizosphere or within the plant. Therefore, because the present study was mainly focused on in vitro antagonistic assays, and although the results are promising, future greenhouse or field validation experiments are needed.

Finally, native or endemic biocontrol agents, such as our isolates of *T. afroharzianum*, offer competitive advantages over non-endemic organisms marketed as biological pesticides. For example, native biocontrol agents are adapted to local biotic and abiotic conditions, making them more effective than commercial products [81]. Furthermore, using native biocontrol agents carries fewer ecological risks, since introducing non-native species that could become invasive can have significant detrimental impacts on the environment and lead to serious consequences not only in the geographic area where they are released but also beyond their initial introduction [82,83]. However, it is essential to optimize formulations to maintain the viability and efficacy of these native biocontrol agents, ensuring they are competitive in quality and practicality and attractive to producers in their region of origin.

## 5. Conclusions

The causal agent of strawberry root and crown rot in the states of Michoacán, Guanajuato, and Baja California, Mexico, is *N. rosae*. According to the PRGI results, all evaluated *T. afroharzianum* strains and *A. cupreus* showed potential as biological control agents against *N. rosae*. Although biological control generally acts more slowly than chemical control, the native isolates exhibited a more consistent inhibition response across all *N. rosae* isolates than the fungicide did. Therefore, the application of biological control agents is recommended as a preventive strategy for disease management. Given the limited exploration of the antifungal activity of the isolates tested in this study (MBP1–MBP4 and IPN 10.0154), further research is needed to evaluate their control efficacy against *N. rosae* in greenhouse or field assays, as well as characterize the mechanisms of action of the evaluated *T. afroharzianum* isolates, including the identification of antifungal metabolites and their potential plant growth-promoting effects on strawberry cultivars. Due to the high variability in the response of *N. rosae* isolates to fungicide treatment, there is a clear need to understand the differential sensitivity of pathogen genotypes to different fungicidal active ingredients used in each strawberry-producing region. Finally, because chemical control remains the main disease management strategy for Mexican strawberry growers, continued research should evaluate additional fungicidal molecules and increase the number of *N. rosae* isolates tested, thereby updating the available information on the efficacy of current fungicides for managing this disease.

## Figures and Tables

**Table 1:** *Neopestalotiopsis* isolates included in the phylogenetic analyses. GenBank accession numbers in bold were generated in this study.

Species	Isolate	Collection Site	Host	GenBank Accession Numbers		
				ITS	*tub2*	*tef1*
*N. australis*	CBS 114159 *	Australia	*Telopea* sp.	KM199348	KM199432	KM199537
*N. clavispora*	MFLUCC 12-0281 *	China	*Magnolia* sp.	JX398979	JX399014	JX399045
*N. cubana*	CBS 600.96	Cuba	Leaf litter	KM199347	KM199438	KM199521
*N. ellipsospora*	MFLUCC 12-0282 *	China	Dead plant materials	JX398980	JX399016	JX399047
	MFLUCC 12-0284	Thailand	Dead plant materials	JX398981	JX399015	JX399046
*N. eucalypticola*	CBS 264.37 *	…	*Eucalyptus globulus*	KM199376	KM199431	KM199551
*N. foedans*	CGMCC 3.9123 *	China	Mangrove plant	JX398987	JX399022	JX399053
	CGMCC 3.9178	China	*Neodypsis decaryi*	JX398989	JX399024	JX399055
*N. formicarum*	CBS 362.72 *	Ghana	Dead Formicidae (ant)	KM199358	KM199455	KM199517
	CBS 115.83	Cuba	Plant debris	KM199344	KM199444	KM199519
*N. honoluluana*	CBS 114495 *	U.S.A.	*Telopea* sp.	KM199364	KM199457	KM199548
*N. iranensis*	CBS 137768 *	Iran	*Fragaria* × *ananassa*	KM074048	KM074057	KM074051
*N. javaensis*	CBS 257.31 *	Indonesia	*Cocos nucifera*	KM199357	KM199437	KM199543
*N. mesopotamica*	CBS 336.86 *	Iraq	*Pinus brutia*	KM199362	KM199441	KM199555
	CBS 299.74	Turkey	*Eucalyptus* sp.	KM199361	KM199435	KM199541
*N. piceana*	CBS 394.48 *	UK	*Picea* sp.	KM199368	KM199453	KM199527
	CBS 254.32	Indonesia	*Cocos nucifera*	KM199372	KM199452	KM199529
	CBS 225.30	…	*Mangifera indica*	KM199371	KM199451	KM199535
*N. rosae*	CBS 101057 *	New Zealand	*Rosa* sp.	KM199359	KM199429	KM199523
	CBS 124745	USA	*Paeonia suffruticosa*	KM199360	KM199430	KM199524
	IPN 10.0227	Michoacán, Mexico	*Fragaria* × *ananassa*	**PX912482**	**PX912159**	**PX912143**
	IPN 10.0228	Michoacán, Mexico	*Fragaria* × *ananassa*	**PX912483**	**PX912160**	**PX912144**
	IPN 10.0229	Michoacán, Mexico	*Fragaria* × *ananassa*	**PX912484**	**PX912161**	**PX912145**
	IPN 10.0230	Michoacán, Mexico	*Fragaria* × *ananassa*	**PX912485**	**PX912162**	**PX912146**
	IPN 10.0231	Michoacán, Mexico	*Fragaria* × *ananassa*	**PX912486**	**PX912163**	**PX912147**
	IPN 10.0232	Michoacán, Mexico	*Fragaria* × *ananassa*	**PX912487**	**PX912164**	**PX912148**
	IPN 10.0233	Michoacán, Mexico	*Fragaria* × *ananassa*	**PX912488**	**PX912165**	**PX912149**
	IPN 10.0234	Michoacán, Mexico	*Fragaria* × *ananassa*	**PX912489**	**PX912166**	**PX912150**
	IPN 10.0235	Michoacán, Mexico	*Fragaria* × *ananassa*	**PX912490**	**PX912167**	**PX912151**
	IPN 10.0236	Michoacán, Mexico	*Fragaria* × *ananassa*	**PX912491**	**PX912168**	**PX912152**
	IPN 10.0237	Michoacán, Mexico	*Fragaria* × *ananassa*	**PX912492**	**PX912169**	**PX912153**
	IPN 10.0238	Michoacán, Mexico	*Fragaria* × *ananassa*	**PX912493**	**PX912170**	**PX912154**
	IPN 10.0239	B. California, Mexico	*Fragaria* × *ananassa*	**PX912494**	**PX912171**	**PX912155**
	IPN 10.0240	B. California, Mexico	*Fragaria* × *ananassa*	**PX912495**	**PX912172**	**PX912156**
	IPN 10.0241	Guanajuato, Mexico	*Fragaria* × *ananassa*	**PX912496**	**PX912173**	**PX912157**
	IPN 10.0242	Guanajuato, Mexico	*Fragaria* × *ananassa*	**PX912497**	**PX912174**	**PX912158**
*N. saprophytica*	CBS 115452 *	China	*Litsea rotundifolia*	KM199345	KM199433	KM199538
*N. steyaertii*	IMI 192475 *	Australia	*Eucalyptus viminalis*	KF582796	KF582794	KF582792
*N. zimbabwana*	CBS 111495 *	Zimbabwe	*Leucospermum cuneiforme*	JX556231	KM199456	KM199545
*P. trachicarpicola*	IFRDCC 2403	China	*Podocarpus macrophyllus*	KC537809	KC537823	KC537816

* = ex-holotype or ex-epitype culture.

**Table 2:** *Trichoderma* isolates included in the phylogenetic analyses. GenBank accession numbers in bold were generated in this study.

Species	Voucher/Culture Nos.	Geographic Origin	Substrate	Genbank Accession Numbers	
				*rpb2*	*tef1*
*T. afarasin* *	CBS 130755 = IMI 393967 = G.J.S. 99-227	Cameroon	Soil	—	AF348093
*T. afroharzianum*	G. Harman 1295-22 = ATCC 29847 = T22 =G.J.S 94-26	Colombia and New York	G. Harman, patented biocontrol strain	—	AF469194
	G.J.S. 00-24	Mexico	Soil	FJ442726	AF443940
	CBS 130439 = G.J.S. 04-02	Montana, USA	Sugar beet	—	FJ463401
	CBS 124620 * = G.J.S. 04-186	Peru	On basidioma of *Moniliophthora roreri* on fruit of *Theobroma*	FJ442691	FJ463301
	CBS 124614 = G.J.S. 04-193	Peru	On basidioma of *Moniliophthora roreri* on fruit of *Theobroma*	FJ442709	FJ463298
	CBS 130443 = G.J.S. 04-197	Peru	On basidioma of *Moniliophthora roreri* on fruit of *Theobroma*	FJ442740	FJ463302
	G.J.S. 05-113	Italy	Wheat seed	—	FJ463378
	CBS 134709 = IBT 41409 = G.J.S. 08-137	—	Ingredient in Canna	—	KP115273
	G.J.S. 97-263	Japan	Soil	—	AF348091
	G.J.S. 97-268	Japan	Soil	—	AF348105
	IMI 393972 = G.J.S. 99-22	Cameroon	Soil	—	AF348106
	MBP-1	Mexico	Soil	**PX922578**	**PX922574**
	MBP-2	Mexico	Soil	**PX922576**	**PX922572**
	MBP-3	Mexico	Soil	**PX922575**	**PX922571**
	MBP-4	Mexico	Soil	**PX922577**	**PX922573**
*T. aggressivum*	CBS 433.95	Northern Ireland	Mushroom compost	FJ442704	AF348097
T. atrobrunneum *	CBS 548.92 = G.J.S. 92-110	France	Decorticated wood of *Fagus* sp.	—	AF443942
T. camerunense *	CBS 137272 = G.J.S. 99-230	Cameroon	Soil	—	AF348107
*T. endophyticum* *	CBS 130729 = IMI 395208 = Dis 217a	Ecuador	*Theobroma gileri* trunk endophyte	—	FJ463319
*T. guizhouense* *	CBS 131803	China	Soil	JQ901400	JN215484
*T. harzianum* *	CBS 226.95	U.K.	Soil	AF545549	AF348101
*T. inhamatum* *	CBS 273.78 = IMI 287526 = G.J.S. 95-39	Colombia	Soil	FJ442725	AF348099
*T. lentiforme* *	CBS 100542 = IMI 393968 = G.J.S. 98-6	French Guiana	Decorticated wood	—	AF469195
*T. lixii* *	CBS 110080 = ATCC MYA-2478 = G.J.S. 97-96 = BPI 745654	Thailand	Decayed *Ganoderma* basidiocarp	—	AF443938
*T. neotropicale* *	G.J.S. 11-185 = LA11	Peru	*Hevea guianensis* trunk endophyte	—	HQ022771
*T. pyramidale* *	CBS 135574 = S73	Italy	*Olea europaea*	KJ665334	KJ665699
*T. rifaii* *	CBS 130746 = Dis 355b	Ecuador	*Theobroma gileri* trunk endophyte	—	FJ463324
*T. simmonsii* *	CBS 130431 = G.J.S. 91-138	USA, Maryland	Decaying bark	FJ442757	AF443935

* = ex-holotype or ex-epitype culture.

**Table 3:** Disease severity (DS) of *Neopestalotiopsis* isolates from inoculated strawberry plants.

Isolate	Origen	DS
IPN 10.0228	Michoacán	90.05 a
IPN 10.0227	Michoacán	86.71 ab
IPN 10.0237	Michoacán	85.05 ab
IPN 10.0231	Michoacán	82.05 ab
IPN 10.0233	Michoacán	80.05 abc
IPN 10.0238	Michoacán	80.05 abc
IPN 10.0232	Michoacán	74.05 abc
IPN 10.0235	Michoacán	70.05 abcd
IPN 10.0236	Michoacán	70.05 abcd
IPN 10.0230	Michoacán	66.71 abcd
IPN 10.0234	Michoacán	60.05 abcde
IPN 10.0241	Guanajuato	58.05 abcde
IPN 10.0229	Michoacán	50.05 bcde
IPN 10.0242	Guanajuato	42.05 cde
IPN 10.0239	B. California	34.05 de
IPN 10.0240	B. California	26.70 e
Control		0.0

DS ranked from highest to lowest. *n* = 6, F = 3.428, df = 15, 48 *p* < 0.001. Means followed by the same letter do not differ significantly.

**Table 4:** Percentage of inhibition of mycelial growth of *Trichoderma afroharzianum* isolates against *Neopestalotiopsis rosae* isolates.

	*Neopestalotiopsis rosae* Isolates															
*T. afroharzianum*	IPN 10.0227	IPN 10.0228	IPN 10.0229	IPN 10.0230	IPN 10.0231	IPN 10.0232	IPN 10.0233	IPN 10.0234	IPN 10.0235	IPN 10.0236	IPN 10.0237	IPN 10.0238	IPN 10.0239	IPN 10.0240	IPN 10.0241	IPN 10.0242
**MBP-1**	76.8 a	79.7 a	75.8 a	73.5 a	77.2 a	78.3 a	74.5 a	75.3 a	75.3 a	73.4 a	77.5 a	51.1 a	70.0 a	50.9 a	82.2 a	81.8 a
**MBP-2**	75.2 a	76.9 a	75.7 a	73.6 a	75.8 a	75.3 a	76.7 a	75.3 a	75.3 a	76.8 a	73.4 ab	52.3 a	73.4 a	44.2 b	75.8 a	84.5 a
**MBP-3**	76.3 a	77.1 a	71.8 a	68.3 a	71.8 a	71.2 a	78.0 a	74.3 a	74.3 a	77.1 a	70.6 b	50.5 a	71.8 a	42.3 b	74.2 a	80.9 a
**MBP-4**	76.0 a	73.3 a	74.7 a	70.0 a	75.7 a	73.5 a	76.7 a	79.7 a	79.7 a	72.7 a	73.6 ab	48.8 a	72.1 a	40.2 b	72.9 a	83.1 a
**Fungicide**	27.2 b	87.2 b	92.6 b	92.8 b	38.9 b	71.2 b	48.4 b	53.3 b	53.3 b	41.2 b	43.1 c	16.6 b	31.5 b	11.8 c	60.4 b	60.3 b

Fungicide = Switch^®^ S62.5WG. Mean ± SE (1.698), *n* = 3, F = 554.070, df = 60, 160 *p* < 0.001. Isolates sharing the same letter in the same column did not show significant differences. Two-way ANOVA and mean separation according to the Holm–Sidak test with a significance level of *p* > 0.05.

**Table 5:** Percentage of inhibition of mycelial growth of *Arcopilus cupreus* on isolates of *Neopestalotiopsis rosae*.

	*Neopestalotiopsis rosae* Isolates															
Isolate	IPN 10.0227	IPN 10.0228	IPN 10.0229	IPN 10.0230	IPN 10.0231	IPN 10.0232	IPN 10.0233	IPN 10.0234	IPN 10.0235	IPN 10.0236	IPN 10.0237	IPN 10.0238	IPN 10.0239	IPN 10.0240	IPN 10.0241	IPN 10.0242
***A. cupreus***	36.7 a	33.3 a	43.3a	30.0 a	40.0 a	40.0 a	50.0 a	20.0 a	56.7 a	40.0 a	43.3 a	16.7 a	30.0 a	10.0 a	60.0 a	60.0 a
**Fungicide**	27.2 b	87.2 b	92.6 b	92.8 b	38.9 a	71.2 b	48.4 b	53.3 b	53.3 b	41.2 a	43.1 a	16.6 b	31.5 a	11.8 a	60.4 a	60.3 a

Fungicide = Switch^®^ S62.5WG. Mean ± 0.04, (F = 24.43, df = 15, *p* < 0.001). Isolated items that do not share the same letter within the same column do not show significant differences, according to the LSD test of the Holm–Sidak test with a significance level of *p* > 0.05.

## Data Availability

The original contributions presented in this study are included in the article. Further inquiries can be directed to the corresponding authors.
